# DOES AGE INFLUENCE IN ENDOSCOPIC THERAPEUTIC SUCCESS ON THE BILIARY TRACT?

**DOI:** 10.1590/0102-672020210003e1607

**Published:** 2022-01-05

**Authors:** Luciano HYBNER, Fernando Issamu TABUSHI, Luis Martins COLLAÇO, Érika Gomes DA ROSA, Bruno de Faria Melquíades DA ROCHA, Mateus Franzoni BOCHNIA

**Affiliations:** 1 Mackenzie Evangelical Faculty of Paraná, Curitiba, PR, Brazil; 2 University Evangelical Mackenzie Hospital, Curitiba, PR, Brazil; 3 Gastrointestinal Endoscopy Service, 9 of July Hospital, São Paulo, SP, Brazil

**Keywords:** Endoscopic retrograde cholangiopancreatography, Endoscopy, Biliary Ducts, Colangiopancreatografia retrógrada endoscópica, Endoscopia, Ductos Biliares

## Abstract

**Background:**

Retrograde endoscopic cholangiopancreatography (ERCP) effectively treats biliary and pancreatic disorders. Its indications are limited and precise, since its misuse delays adequate treatment, increases costs and to patient´s adverse events.

**Aim:**

To compare clinical, radiological and exploratory characteristics in relation to therapeutic success in patients undergoing ERCP in relation to age.

**Method:**

421 patients who underwent the method were retrospectively studied; those who were not able to access the duodenal papilla were excluded. The patients were divided into two age groups: <60 years (group 1) and >60 years (group 2), and the variables of gender, examination indications, radiological findings, therapeutic success, diagnosis and the occurrence of immediate adverse events were analyzed.

**Results:**

177 patients were allocated to group 1 and 235 to group 2. The main indication found in both groups was choledocholithiasis. In group 2, the number of cases of acute cholangitis (p=0.001), biliary stenosis (p=0.002) and papilla cancer (p=0.046) was higher. In this group, urgent indication for ERCP was higher (p=0.042), as well as the diagnosis of biliary tract dilatation (p<0.001). The placement of prostheses was the most common procedure performed in both groups, but the greatest number of patients in absolute quantity occurred in group 2. In group 1, the success in catheterization and the chance of achieving clearing of the biliary tract was significantly higher in compared to group 2 (p=0.016, OR=2.1).

**Conclusion:**

The success of catheterization and complete clearance of the bile duct was significantly higher in the group of young patients.

## RESUMO

**Racional:**

A colangiopancreatografia endoscópica retrógrada (CPRE) trata eficazmente afecções biliares e pancreáticas. Suas indicações são limitadas e precisas, uma vez que seu uso indevido atrasa o tratamento adequado, aumenta os custos e submete pacientes a eventos adversos.

**Objetivo:**

Comparar as características clínicas, radiológicas e exploratórias em relação ao sucesso terapêutico em pacientes submetidos à CPRE com relação à idade.

**Método:**

Foram estudados retrospectivamente 421 pacientes submetidos ao método sendo excluídos aqueles onde não foi possível ao acesso a papila duodenal. Os pacientes foram divididos em dois grupos etários: <60 anos (grupo 1) e >60 anos (grupo 2) e analisadas as variáveis gênero, indicações do exame, achados radiológicos, sucesso terapêutico, diagnóstico e a ocorrência de eventos adversos imediatos.

**Resultados:**

177 pacientes foram alocados no grupo 1 e 235 no grupo 2. A principal indicação encontrada em ambos os grupos foi coledocolitíase. No grupo 2, o número de casos de colangite aguda (p=0,001), estenose biliar (p=0,002) e neoplasia de papila (p=0,046) foi superior. Nesse grupo a indicação da CPRE em caráter de urgência foi superior (p=0,042), bem como o diagnóstico de dilatação da via biliar (p<0,001). A colocação de próteses foi o procedimento mais comumente realizado nos dois grupos, mas o maior número de doentes em quantidade absoluta ocorreu no grupo 2. No grupo 1, o sucesso na cateterização e a chance de conseguir o clareamento da via biliar foi significativamente superior em relação ao grupo 2 (p=0,016, OR=2,1).

**Conclusão:**

O sucesso da cateterização e o clareamento completo da via biliar foi significativamente superior no grupo de pacientes jovens.

## INTRODUCTION

Endoscopic retrograde cholangiopancreatography (ERCP) was first described in 1968 as a method to assess the biliary and pancreatic pathway^9^. Since 1974, with the description of endoscopic sphincterotomy, its therapeutic capacity has evolved a lot^7^. It is a complex endoscopic procedure, with great potential for the treatment of biliary and pancreatic disorders and indicated in obstructive jaundice, caused by choledocholithiasis, benign or malignant stenosis. Biliary fistulas, hepatopancreatic ampoule sphincter dysfunction (Oddi), ampular tumor, recurrent acute pancreatitis, chronic pancreatitis, pancreatic duct fistula or even in the treatment of fluid collections may be attributions for the treatment by this procedure^3^.

With the evolution of less invasive imaging methods such as transcutaneous ultrasonography, computed tomography, magnetic resonance cholangiopancreatography and endoscopic ultrasonography, there is currently a tendency to indicate ERCP only for therapeutic purposes^1^.

Acute cholangitis is considered the most consistent indication for first-line treatment with ERCP in cases of suspected biliary obstruction or extrahepatic stenosis. Selective cannulation of the biliary tree is successful in up to 90% of cases of choledocholithiasis and should be selectively indicated, as its unnecessary use delays adequate treatment, increases costs and submits patients to adverse events^9^.

The aim of this study was to compare the clinical, radiological and exploratory characteristics in relation to therapeutic success in patients undergoing ERCP under 60 and elderly over 60 years.

## METHOD

This is a retrospective, cross-sectional, observational study based on a review of medical records of 421 patients undergoing ERCP at the Endoscopy Service of 9 of July Hospital, São Paulo, SP, Brazil. Patients whose access to the second duodenal portion and, consequently, to the greater duodenal papilla, was not possible, were excluded. The others were divided into two groups: age <60 years (G1) and >60 years (G2), according to the classification established by the World Health Organization for elderly patients in developing countries. Indications for ERCP, radiological findings, therapeutic success, diagnosis and immediate complications were documented.

### Statistical analysis

It was initially made descriptively through absolute and relative frequencies (percentage). The inferential analyzes employed in order to confirm or refute evidence found in the descriptive analysis were the Pearson’s chi-square test or Fisher’s exact test when comparing the two groups, G1 and G2. They were analyzed according to gender, indications for ERCP, radiological findings, therapeutic success, diagnosis and immediate complications. In all conclusions through inferential analysis, a significance level equal to 5% was used.

## RESULTS

**FIGURE 1:**
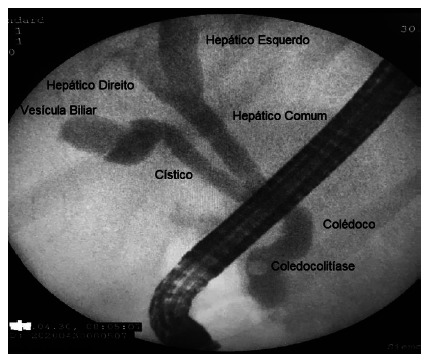
Single stone choledocholithiasis visualized by ERCP in the distal portion

The selected sample consisted of 421 patients undergoing ERCP (Figure 1). Patients unable to access the papilla were excluded. After exclusion, 412 patients were selected and divided into two age groups, above and below 60 years.

### Patients aged <60 years (Group 1 - G1)

This group consisted of 177 patients under 60 years of age; there were 114 (64.4%) women and 63 (35.6%) men. ERCP was indicated for diagnosis in six (3.4%) and therapeutic in 171 (96.6%). Twenty-one (11.9%) performed ERCP on an emergency basis and 156 (88.1%) electively.

Considering the main indications, 107 (60.5%) had previously documented choledocholithiasis, five (2.8%) cholangiocarcinoma, three (1.7%) tumor of the greater papilla of the duodenum, 16 (9.0%) acute pancreatitis and two (1.1%) complications of chronic pancreatitis. Acute cholangitis was the cause in four (2.3%), while obstructive jaundice and fistula in 18 (10.2%) and four (2.3%), respectively. Three (1.7%) underwent ERCP for indication of malignant stenosis and 16 (9.0%) for benign stenosis.

Considering the radiological presentation, 52 (31%) had a dilated bile duct, 121 (72.5%) had calculus in the bile duct, and 28 (16.7%) stenosis identified by ERCP. Success rate during selective catheterization was 95%, infundibulotomy was used in 8.5% of cases. In 171 (96.6%) sphincterotomy was performed; in 30 (16.9%) plastic prosthesis placement was done, in four (2.3%) self-expanding metallic prosthesis insertion and in three endoscopic papillectomy. Adverse events occurred during the examination in three (1.7%) cases: bleeding (n=2) and perforation (n=1) case. All were treated conservatively. The success rate for clearing the biliary tree was possible in 162 (91.5%) patients in this group.

### Patients aged over 60 years (Group 2 - G2)

A total of 235 patients were included, 121 (51.5%) women. ERCP was indicated for treatment in 232 (98.7%). Forty-three (19.4%) performed it urgently and 179 (80.6%) electively. The main indications in this group were 107 (45.5%) for previously documented choledocholithiasis, 10 (4.3%) for cholangiocarcinoma, 13 (5.5%) for tumors of the greater papilla of the duodenum, 13 (5.5%) for acute pancreatitis and two (0.9%) for complications of chronic pancreatitis. Acute cholangitis and obstructive jaundice were reasons, each, in 26 (11.1%) patients. Twenty-three (9.8%) underwent ERCP for indication of malignant stenosis and 14 (6.0%) for benign.

Considering the radiological presentation, 129 (57.1%) had a dilated bile duct, 133 (63.9%) had gallstones in the bile duct, and 62 (29.4%) confirmed the presence of previously suspected stenosis.

Catheterization success occurred in 210 (89.4%), and infundibulotomy was used for access on 22 (9.7%) occasions. Five (2.1%) had adverse events during the exam: perforation (n=2, 0.9%), bleeding (n=2, 0.9%) and migration of the prosthesis to the common bile duct (n=1, 0.4%). One patient had a perforation and was operated on, dying after 15 days. A total of 232 (98.7%) underwent sphincterotomy, and in 68 (30.4%) a plastic prosthesis was placed, in 12 (5.4%) a metallic prosthesis and in four (1.8%) papillectomy. Clearing of the biliary tree was possible in 196 (83.4%) patients.

### Comparison between the two groups

When comparing the two groups, it was observed that there was a predominance of women in both, but in G1 it was higher, with a statistically significant difference (p=0.009). Regarding the nature of the indication for the examination, there was a predominance of procedures performed electively, but in G1, those considered as urgent had an even lower frequency (p=0.042, Table 1). In both groups, almost all were performed therapeutically, with no significant difference between them (p=0.181, Table 1).

**TABLE 1:** Pre-ERCP characteristics

Indications	Group 1	Group 2	p
Sex F/M	114/63	121/114	0.009
Therapeutics/diagnosis	171(96.6%) / 6 (3.4%)	232 (98.7%) / 3 (1.3%)	0.181
Urgency/elective	21 (11.9%) / 156 (88.1%)	21 (11.9%) / 156 (88.1%)	0.042

The inferential results revealed that the age groups had the same profile regarding indications for cholangiocarcinoma (p=0.443), acute pancreatitis (p=0.168), complications of chronic pancreatitis (p>0.999) and obstructive jaundice (p=0.771). Patients in G1 had more indications for choledocholithiasis (p=0.003) and biliary fistula (p=0.033), while those in G2 for neoplasm of the greater papilla of the duodenum (p=0.046), acute cholangitis (p=0.001) and malignant biliary stenosis (p=0.002, Table 2).

**TABLE 2:** Indications for carrying out the ERCP

Most frequent indications	Group 1	Group 2	p
Choledocolithiasis	107 (60.5%)	107 (45.5%)	0.003
Obstructive jaundice	18 (10.2%)	26 (11.1%)	0.771
Benign stenosis	16 (9%)	14 (6.0%)	0.999
Acute pancreatitis	16 (9%)	13 (5.5%)	0.168
Cholangiocarcinoma	5 (2.8%)	10 (4.3%)	0.443
Acute cholangitis	4 (2.3%)	26 (11.1%)	0.001
Fistula	4 (2.3%)	0	0.033
Papilla neoplasm	3 (1.7%)	13 (5.5%)	0.046
Malignant stenosis	3 (1.7%)	23 (9.8%)	0.002
Chronic pancreatitis	2 (1.1%)	2 (0.9%)	0.999

As for the radiological presentation, in G2 there was a greater finding of calculi, although without statistical significance. However, significantly higher was the frequency of dilated bile duct (p<0.001) and confirmed stenosis (p=0.004, Table 3).

**TABLE 3:** Radiological presentation during the procedure

	Group 1	Group 2	p
Calculi	121 (72.5%)	133 (63.9%)	p=0.08
Dilated bile duct	52 (31%)	129 (57.1%)	p<0.001
Stenosis	28 (16.7%)	62 (29.4%)	p=0.004

Success in catheterization (p=0.043) and clearance of the bile duct (p=0.016) were obtained less frequently in G2 when compared to G1, with Odds-ratio (OR) in relation to bile duct clearance of 2.1 (Table 4).

**TABLE 4:** CPRE results

	Group 1	Group 2	p
Catheterization success	168 (94.9%)	210 (89.4%)	p=0.043
Bile duct clearing	162 (91.5%)	196 (83.4%)	p=0.016

The most frequently performed procedures in both groups, excluding papilotomy, were placement of both plastic and metallic prostheses, but with a higher frequency (p=0.001) in G2 (Table 5).

**TABLE 5:** Procedures performed

	Group 1	Group 2	p
Papillectomy	3 (1.7%)	4 (1.8%)	p>0.999
Plastic prosthesis	30 (16.9%)	68 (30.4%)	p=0.001
Metal prosthesis	4 (2.3%)	12 (5.4%)	p=0.001

Regarding immediate complications inherent to the procedure, there was no statistically significant difference (p>0.999).

## DISCUSSION

The present study, despite being retrospective, manages to analyze the characteristics of the procedures performed by a multidisciplinary team, in a single tertiary center. As it is a national reference, the selection bias may be present at the time when more complex cases were referred to this service.

Although ERCP is one of the most invasive endoscopic procedures, with a high rate of adverse events compared to other endoscopic procedures, it is considered safe even among the elderly^4^. The main risk factors for post-ERCP complications in this group are the presence of chronic obstructive pulmonary disease and difficult cannulation^12^. Immediate complications were low in both groups, with no statistically significant difference between them.

In parallel to the increase in life expectancy both nationally and worldwide^11^ and the increase in the incidence of biliopancreatic diseases in this population, the performance of ERCP in the elderly has become increasingly frequent because they bring comorbidities inherent to age, such as systemic arterial hypertension, diabetes mellitus, coronary artery disease^13^. Consequently, the performance of ERCP, which is recognized for having lower morbidity and mortality compared to surgical treatment, has been increasingly indicated as a treatment for these biliary and pancreatic diseases.

The procedure most frequently performed in both groups, especially in G2, was the placement of prostheses, both plastic and metallic. It has specific indications, such as in cases where there was no complete extraction of the stones, in the absence of adequate biliary drainage at the end of the procedure^2^, in stenoses or fistulas.

The success rate in accessing the biliary tract and its clearance at the end of the procedure found in this study was higher in G1, but in G2 the value around 90% is considered high, confirming the effectiveness of the procedure also among the elderly and going to the meeting the provisions in the literature^10^. Difficulty in cannulating the biliary tract may be more frequent in the elderly due to anatomical changes resulting from previous operations, higher rate of duodenal diverticulum or due to changes in the papilla resulting from the passage of stones through it^6^.

ERCP is an endoscopic procedure that can be used for both diagnosis and therapy; but, due to the advancement of other diagnostic methods and their complexity, following the current trend, it is being used mainly for the treatment of biliopancreatic diseases.

The main indication found in this study in both groups was choledocholithiasis, but it is noteworthy that in group G2, there was a greater indication for acute cholangitis, biliary stenosis and papilla cancer. In these patients, bile duct stenosis and dilatation were also more frequently diagnosed.

## CONCLUSION

In the group with younger patients, success in catheterization and the chance of achieving clearance of the biliary tree are significantly higher than in older patients.
